# Sirtuins as Molecular Mediators of Caloric Restriction in the Pancreas: Implications for β-Cell Function, Metabolism, and Longevity

**DOI:** 10.3390/ijms27156922

**Published:** 2026-08-01

**Authors:** Katarzyna Zgutka, Wioletta Mikołajek-Bedner, Kamila Szumilas, Maciej Tarnowski

**Affiliations:** 1 Department of Physiology in Health Sciences, Faculty of Health Sciences, Pomeranian Medical University, 71-210 Szczecin, Poland; katarzyna.zgutka@pum.edu.pl; 2 Department of Obstetrics and Gynecology, Pomeranian Medical University, 70-111 Szczecin, Poland; wioletta.mikolajek.bedner@pum.edu.pl; 3 Department of Physiology, Pomeranian Medical University, 70-111 Szczecin, Poland; kamila.szumilas@pum.edu.pl

**Keywords:** pancreatic β-cells, caloric restriction, sirtuins, aging, insulin secretion, metabolic homeostasis

## Abstract

Caloric restriction (CR), defined as a 30–60% decrease in ad libitum food intake without malnutrition, has emerged as one of the most robust non-pharmacological interventions for promoting metabolic health and longevity in various species, including yeast, worms, flies, rodents, and perhaps non-human primates. In addition, CR has been shown to reduce the incidence of age-related disorders (for example, diabetes, cancer, and cardiovascular disorders) in mammals. Among the key organs influenced by CR, the pancreas—particularly the insulin-producing β-cells—plays a central role in maintaining glucose homeostasis and metabolic balance. A growing body of evidence suggests that CR exerts its beneficial effects, at least in part, through the modulation of nutrient-sensing pathways and epigenetic regulators. Sirtuins, a family of NAD^+^-dependent deacetylases and ADP-ribosyltransferases, have gained attention as pivotal molecular mediators of CR. By responding to changes in cellular energy status, sirtuins regulate diverse processes including gene expression, oxidative stress response, mitochondrial function, and autophagy. In the pancreas, sirtuins such as SIRT1, SIRT3, and SIRT6 have been implicated in preserving β-cell function, enhancing insulin secretion, and protecting against metabolic stress and inflammation. This review critically examines current evidence regarding the role of individual sirtuins in mediating the pancreatic response to caloric restriction, with particular emphasis on β-cell physiology, insulin secretion, mitochondrial function, autophagy, oxidative stress, and inflammatory signaling. We further discuss how these molecular mechanisms contribute to systemic metabolic homeostasis and may influence healthy longevity. Importantly, we integrate experimental findings with emerging clinical evidence demonstrating the recovery of β-cell function following dietary energy restriction and identify current controversies, limitations, and key knowledge gaps that should guide future translational research. Collectively, available evidence suggests that sirtuins represent central molecular links between caloric restriction and β-cell adaptation, highlighting their potential as therapeutic targets for preserving pancreatic function and preventing metabolic disease.

## 1. Introduction

The pancreas plays a central metabolic role in human physiology, encompassing both exocrine and endocrine functions. The exocrine compartment produces and releases pancreatic juice enriched with a spectrum of digestive enzymes that facilitate the breakdown of carbohydrates, proteins, and lipids in the gastrointestinal tract [1]. The endocrine compartment, composed of specialized islet cells, secretes hormones directly into the circulation, with insulin from pancreatic β-cells being central to the regulation of glucose homeostasis [1]. Following the ingestion of nutrients, elevations in circulating glucose serve as a primary stimulus for β-cell insulin secretion [2]. Glucose-induced insulin secretion (GSIS) is a physiological process by which the pancreas releases insulin in response to an increase in blood glucose levels [3]. When glucose enters the bloodstream after a meal, it is taken up by β-cells in the pancreas through glucose transporters, primarily GLUT2 in rodents and GLUT1/3 in humans [4]. Once inside the β-cells, glucose is phosphorylated through glucokinase into glucose-6-phosphate [4]. The metabolism of glucose via glycolysis and the Krebs cycle generates ATP. The mitochondrial inner membrane protein ATP/ADP translocase exchanges mitochondrial ATP, produced by oxidative phosphorylation, for cytosolic ADP. This leads to an increase in the cytosolic ATP/ADP ratio [5]. The resting membrane potential of the pancreatic β-cell is determined by the ATP-sensitive potassium (K^+^ATP) channel, which in turn has been shown to result in depolarization of the plasma membrane [6]. These processes lead to the activation of voltage-dependent L-type Ca^2+^ channels, thereby triggering Ca^2+^ influx. The increase in intracellular Ca^2+^ concentration facilitates the priming and fusing of insulin granules to the plasma membrane, and insulin exocytosis follows [6]. Once released, insulin promotes glucose uptake in skeletal muscle and adipose tissue, suppresses hepatic gluconeogenesis, and stimulates glycogen synthesis in the liver, thereby lowering postprandial glucose levels and preventing hyperglycemia [2].

Importantly, mitochondria in β-cells are not simply energy factories but are central integrators of metabolic coupling signals. Recent work performed by Rivera Nieves AM et al. [7] highlights that mitochondria in β-cells serve multiple roles: they link glucose flux to ATP production, generate metabolic coupling factors (e.g., NADH, malate, citrate, ROS) that amplify insulin release beyond the ATP-triggered pathway, and regulate redox state, calcium handling, and organellar dynamics (fission/fusion, biogenesis, mitophagy), all of which are critical for proper secretory function [7]. Under conditions of metabolic stress—such as chronic hyperglycemia, high free fatty acids (lipotoxicity), oxidative stress, or mitochondrial dysfunction—β-cells exhibit impaired GSIS, reduced mitochondrial ATP production, disturbed redox homeostasis, and an increased probability of apoptosis or dedifferentiation. For instance, impaired mitochondrial dynamics (excessive fission or loss of fusion) in β-cells is linked to defective insulin secretion and β-cell death [7,8].

Various nutritional strategies reported in the literature have been shown to influence pancreatic β-cell function [3,9,10,11,12,13]. Chronic exposure to a high-fat diet results in an accumulation of circulating free fatty acids (FFAs), which can exert lipotoxic effects on β-cells [14]. These effects are further exacerbated by increased adiposity, which promotes a chronic low-grade inflammatory state. Elevated levels of pro-inflammatory cytokines in the circulation can trigger β-cell apoptosis, progressively impairing insulin secretion capacity. This sequence of events contributes to the pathogenesis of β-cell dysfunction, a hallmark of type 2 diabetes mellitus (T2DM). Indeed, obesity and sustained high-fat feeding have been strongly associated with both β-cell failure and the clinical onset of T2DM. Kitabchi et al. [13] reported significant advantages of a 6-month hypocaloric high-protein diet versus a hypocaloric high-carbohydrate diet on markers of β-cell function, insulin sensitivity, oxidative stress, lipid peroxidation, proinflammatory cytokines, and adipokines in normal, obese females without diabetes [13].

Calorie restriction without a lack of essential nutrients is the most potent non-pharmacological and non-genetic experimental intervention to attenuate aging and prevent chronic metabolic diseases [15]. Throughout the literature, the term CR is often used interchangeably with dietary restriction (DR), referring to a reduction of 20–60% of energy intake while maintaining proper levels of minerals and vitamins necessary for the body’s proper functioning [16]. Some researchers utilizing animal models applied mild caloric restriction—approximately 10–15%—which has also been shown to produce notable biological effects [17,18]. The groundbreaking study that first linked energy intake to health and longevity in rats was published by McCay et al. in 1935 [19]. Although research on the effects of caloric restriction (CR) continued over the following five decades, it was not until the 1980s that its influence on the aging process was fully recognized. Since then, the impact of CR on health and aging-related phenotypes has been extensively investigated in invertebrate models, laboratory rodents, and non-human primates [19]. It is known that CR induces a reduction in energy expenditure that is larger than the loss of metabolic mass, i.e., fat-free mass and fat mass. According to prevailing theories of mammalian aging, this disproportionate reduction in metabolic rate, known as metabolic adaptation, reduces oxidative damage and thereby delays age-associated declines in physiological function [15]. Under CR or low-energy states, the cellular flux through glycolysis and the tricarboxylic acid (TCA) cycle slows down, reducing NADH production and shifting the mitochondrial redox balance in favor of NAD^+^ over NADH [20]. Concurrently, CR activates energy-sensing kinases such as AMP-activated protein kinase (AMPK), which upregulate NAD^+^ biosynthetic enzymes such as nicotinamide phosphoribosyltransferase (NAMPT), thereby promoting NAD^+^ salvage and replenishing the NAD^+^ pool [20,21]. It is known that the elevated NAD^+^/NADH ratio under CR thus enhances the activity of sirtuins (SIRTs), enabling them to deacetylate histones and various transcriptional regulators, modulate mitochondrial proteins, and facilitate adaptations in metabolism, proteostasis, and stress resistance [2,20,22,23]. In pancreatic β-cells, this NAD^+^–sirtuin axis likely represents a key molecular link by which CR translates systemic energy limitation into cell-intrinsic adaptations that favor β-cell longevity and functional stability. While direct in vivo data remain scarce, several lines of evidence support this model [24,25]. For example, activation of sirtuins has been shown to improve glucose-stimulated insulin secretion and to modulate insulin signaling in peripheral tissues, thereby reducing metabolic stress on β-cells [24]. Moreover, maintenance of NAD^+^ homeostasis appears critical for preserving β-cell function under metabolic or age-related stress. In a recent study, mice lacking functional nicotinamide riboside kinase 1 (NRK1)—an enzyme required for the utilization of the NAD^+^ precursor nicotinamide riboside (NR)—exhibited impaired β-cell response (glucose intolerance, reduced β-cell function) when exposed to a high-fat diet or aging, underscoring the importance of NAD^+^ salvage pathways in β-cell resilience [25]. These findings collectively suggest that NAD^+^ availability and sirtuin activity may constitute a mechanistic hub aligning nutrient status with β-cell metabolic programming. Through NAD^+^-mediated activation of sirtuins, CR could promote mitochondrial efficiency, enhance resistance to oxidative and metabolic stress, support proteostasis and autophagy, and consequently preserve β-cell identity and function over time.

This literature review aims to evaluate the current state of knowledge on the role of individual sirtuins in mediating the pancreatic response to caloric restriction, with particular emphasis on β-cell physiology, insulin secretion, mitochondrial function, autophagy, oxidative stress, and inflammatory signaling. To achieve this, major scientific databases such as PubMed, Scopus, and Web of Science were searched between June 2025 and May 2026 using calorie restriction, sirtuin 1, 2, 3, 4, 5, 6, 7, and pancreatic β cells as the primary keywords. The aim was to identify relevant studies to provide a comprehensive narrative overview of the current evidence.

## 2. Impact of Calorie Restriction on β-Cell Physiology and Homeostasis

**Figure 1:**
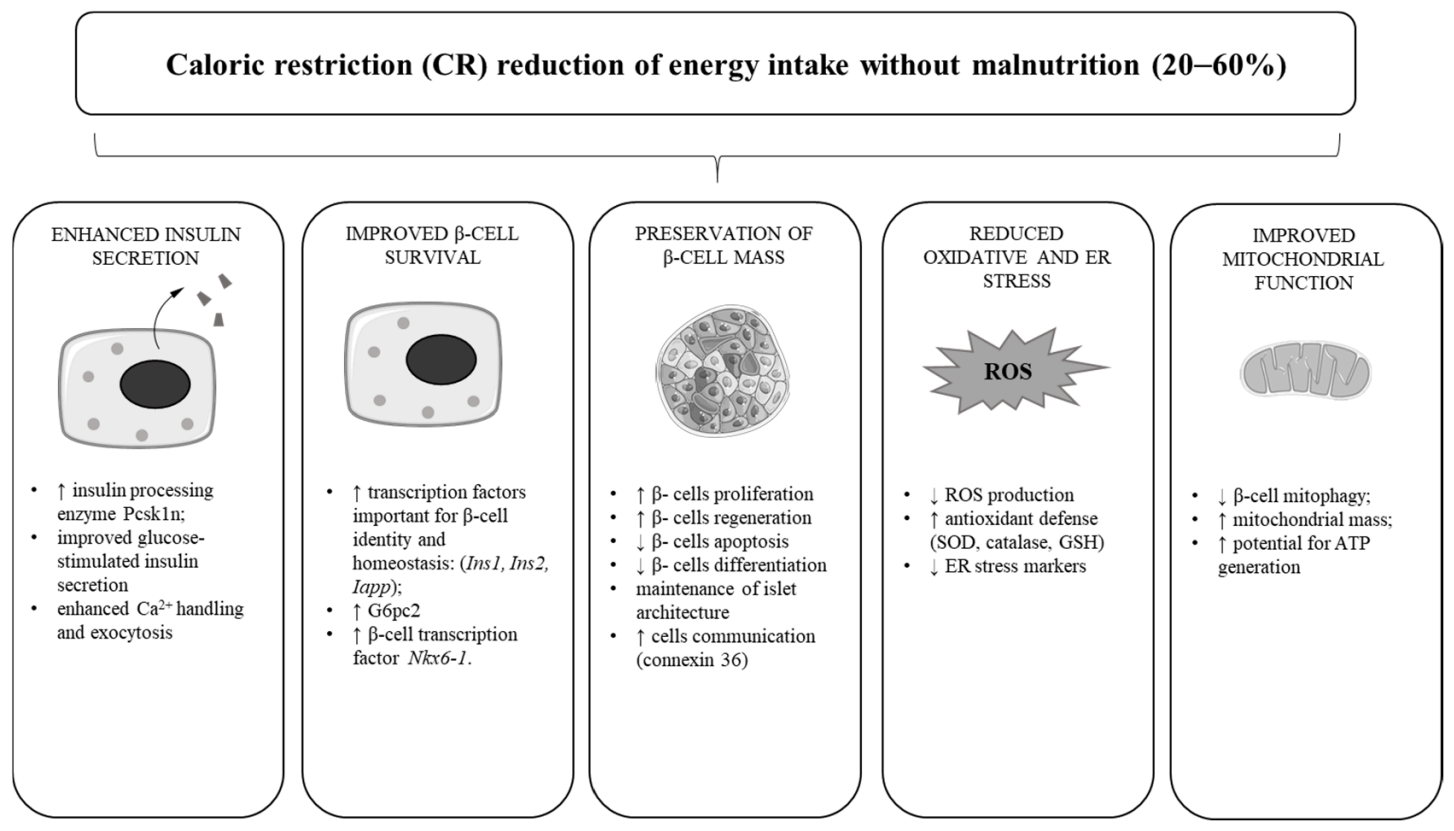
Impact of caloric restriction on β-cell physiology and homeostasis (animal/cellular models), ↑ increase, ↓ decrease. Created by the authors using Microsoft PowerPoint with graphical elements from Servier Medical Art (https://smart.servier.com), licensed under the Creative Commons Attribution 4.0 International License (CC BY 4.0).

Previously, studies strongly supported the clinical implication of CR in preventing β-cell dysfunction and delaying the onset of diabetes or impaired glucose tolerance during the aging process [23,26]. Conversely, caloric restriction has been shown to protect β-cell integrity during the development and progression of T2DM [23]. Experimental evidence indicates that CR mitigates lipotoxic and inflammatory stress, preserves insulin secretory capacity, and maintains the glucose responsiveness of β-cells [27,28]. This protective effect of CR not only delays β-cell failure but also supports systemic glucose homeostasis under conditions of metabolic stress [27]. Figure 1 provides an overview of the pleiotropic effects of caloric restriction on pancreatic β-cell physiology and homeostasis in animal models, highlighting the interconnected roles of mitochondrial metabolism, oxidative stress, autophagy, inflammation, and insulin secretory function.

Collectively, these findings emphasize that pancreatic β-cells are central regulators of glucose balance and that their dysfunction has far-reaching consequences, leading to severe metabolic disturbances, including T2DM. The study by Peter et al. [29] investigated the effects of a 4week low-calorie dietary intervention (~1500 kcal/day) on pancreatic β-cell function in 23 overweight, normoglycemic adults. The intervention resulted in an average weight loss of approximately 3.5%, with significant reductions in trunk and visceral fat. Most notably, β-cell function, as measured by the dynamic disposition index (which integrates insulin secretion relative to insulin sensitivity), increased by 128% after the intervention. Robust regression analysis demonstrated a significant association between weight loss and improvement in β-cell function, with an estimated 23% increase in the disposition index per 1% reduction in body weight [29].

dos Santos et al. [30] investigated how mouse β-cells adapted during 2 months of 20% CR, and whether this intervention could delay the hallmarks of β-cell aging. Using a gene regulatory network analysis, they revealed that CR upregulates transcription factors important for β-cell identity and homeostasis, including both insulin genes (*Ins1*, *Ins2*), amylin (*Iapp*), the insulin processing enzyme Pcsk1n, the glucose-6 phosphatase enzyme G6pc2, and the β-cell transcription factor *Nkx6-1*. Moreover, imaging metabolomics has demonstrated that β-cells under CR are more energetically competent. In fact, high-resolution microscopy has shown that CR reduces β-cell mitophagy by increasing mitochondrial mass and the potential for ATP generation [30]. Mechanistically, CR improves the quality-control systems of the β-cell, enhancing autophagy and maintaining organelle integrity, which collectively mitigate oxidative and endoplasmic reticulum stress [31].

Recent findings imply a potential role for CR in intercellular communication within the islets of Langerhans mediated by connexin36 (Cx36) gap junctions [32]. It has been shown that 1 month of 40% caloric restriction protects against obesity-induced decreases in gap junction coupling and preserves glucose-stimulated calcium signaling, including Ca^2+^ oscillation coordination and oscillation amplitude in C57BL6 mice. Moreover, CR also promotes a slight increase in glucose metabolism by recovering glucose-stimulated insulin secretion [32].

It has also been found that at the level of stimulus–secretion coupling, CR alters intracellular Ca^2+^ dynamics, producing shorter and faster oscillations and modifying the connectivity and synchrony of the islet network [33]. Such changes likely reflect adaptive remodeling of IP_3_ receptor signaling, ER–mitochondria communication, and cAMP pathways under reduced nutrient load [33]. These effects are complemented by CR-driven reductions in inflammatory signaling and lipotoxic stress, both of which are key contributors to β-cell dedifferentiation and dysfunction in metabolic disease [28]. In db/db mouse models, long-term CR significantly reduces β-cell dedifferentiation, restoring the expression of lineage-specific genes such as *Pdx1*, *Glut2*, *Nkx6.1*, and *Mafa*, and reactivating GLP-1 signaling pathways, thereby preserving β-cell identity and function [28].

Moreover, modulation of diet composition during CR influences dedifferentiation: high-fat/low-carbohydrate CR suppresses β-cell dedifferentiation compared to high-carbohydrate CR regimens [27].

A recent study performed by Luo et al. [34] demonstrated the effects of both a GLP-1 receptor agonist (semaglutide) and calorie restriction on pancreatic islet morphology, β-cell function, and gut microbiota composition in high-fat diet (HFD)-induced obese mice. Although the research did not directly investigate the isolated effects of calorie restriction on pancreatic β-cells, it clearly demonstrated that the application of an energy-limiting intervention can significantly reduce fasting hyperglycemia and HOMA-IR, indicating improvements in glucose tolerance and insulin sensitivity. Histological analyses revealed that HFD-induced islet enlargement and β-cell hyperplasia were attenuated by CR. Apoptosis in β-cells was suppressed, and proliferation increased, suggesting structural remodeling and enhanced regenerative capacity under CR interventions. Finally, this study documented an increased expression of key regulators such as METTL3/14, PDX-1, and components of the insulin signaling cascade (AKT-1, INSR, IRS-2, IGF1R) in CR-treated mice. These changes were associated with normalized *m6A* methylation levels in pancreatic islets, implicating epitranscriptomic regulation in islet functional recovery [34].

However, accumulating evidence indicates that the beneficial effects of GLP-1 receptor agonists extend far beyond weight reduction alone. Although body weight loss substantially improves insulin sensitivity and contributes to better glycemic control, GLP-1 receptor agonists exert numerous weight-independent actions, including direct stimulation of glucose-dependent insulin secretion, suppression of glucagon release, preservation of β-cell identity and function, attenuation of endoplasmic reticulum stress, reduction in oxidative stress, and inhibition of β-cell apoptosis [35,36]. Moreover, GLP-1 signaling involves both central and peripheral neural pathways, particularly through vagal afferents and autonomic nervous system circuits that coordinate communication between the gastrointestinal tract, pancreas, liver, and brain, thereby regulating appetite, gastric emptying, and glucose homeostasis [37,38]. Consequently, the metabolic improvements observed following GLP-1 receptor agonist therapy are likely to result from the integration of direct pancreatic effects, indirect neuroendocrine mechanisms, and systemic metabolic adaptations rather than from weight loss alone. In addition, GLP-1 receptor agonists have been shown to exert anti-inflammatory and cytoprotective effects by modulating inflammatory signaling pathways, improving mitochondrial function, and reducing cellular oxidative stress. These pleiotropic actions may partially overlap with molecular pathways regulated by sirtuins, particularly SIRT1 and SIRT3, which are recognized as important regulators of mitochondrial homeostasis, cellular metabolism, oxidative stress responses, and β-cell survival. Therefore, when interpreting studies evaluating GLP-1 receptor agonists, it is important to consider that their therapeutic efficacy reflects the combined contribution of weight loss-dependent and weight loss-independent mechanisms.

In a Fischer 344 rat model, long-term CR was shown to preserve autophagic function in pancreatic islets during aging, counteracting age-associated declines observed in rats fed an ad libitum or high-fat diet. Aging and high-fat feeding decreased the β-cell-to-islet area ratio, disrupted islet architecture, increased markers of cellular senescence, and reduced the expression of key autophagy-related proteins, consistent with compromised autophagic flux and the accumulation of p62 and polyubiquitin aggregates. In contrast, 24-month-old rats maintained on a CR regimen exhibited an islet autophagy profile closely resembling that of young (3-month-old) controls, indicating that CR delays the age-related decline in islet autophagy and mitigates the accumulation of damaged proteins and organelles, effects that are likely relevant for maintaining β-cell homeostasis and function with age [39].

The strongest clinical evidence supporting the beneficial effects of caloric restriction on pancreatic β-cell function comes from the pioneering work of Roy Taylor and colleagues, who fundamentally changed the understanding of type 2 diabetes as a potentially reversible metabolic disorder rather than an inevitably progressive disease [40]. Using a very-low-calorie diet (VLCD), they demonstrated that substantial energy restriction rapidly decreases ectopic fat accumulation in both the liver and pancreas, leading to normalization of hepatic insulin sensitivity within days and the gradual restoration of β-cell function over subsequent weeks. Remarkably, the recovery of first-phase insulin secretion closely paralleled the reduction in pancreatic fat content, suggesting that β-cell dysfunction is, at least in part, a reversible consequence of metabolic overload rather than irreversible cell loss.

These findings were subsequently confirmed in the DiRECT trial, where sustained weight loss achieved through intensive dietary intervention resulted in long-term remission of type 2 diabetes in a substantial proportion of participants. Importantly, remission was associated with the persistent recovery of β-cell function and maintenance of reduced pancreatic fat, emphasizing that restoration of β-cell physiology represents a central mechanism underlying diabetes reversal rather than merely improved peripheral insulin sensitivity [41]. Although these landmark studies did not directly investigate sirtuin signaling, they provide an important physiological framework for understanding the molecular adaptations induced by caloric restriction.

## 3. Activity of Sirtuins in Pancreatic β Cells—Molecular Mechanisms

Sirtuins are linked to diet and metabolism through their dependence on the availability of the cofactor NAD^+^. To date, seven proteins belonging to the sirtuin family (SIRT1–SIRT7) have been identified in mammals [42] and are distributed across different cellular compartments. Sirtuin 2 is mainly present in the cytoplasm; sirtuins 3, 4, and 5 predominate in the mitochondria, whereas sirtuins 1, 6, and 7 are localized in the cell nucleus [42].

Sirtuins represent a class of enzymes that are pivotal for the process of histone deacetylation and the posttranslational modification of proteins. Consequently, they can regulate a wide spectrum of biological processes, including glucose and lipid metabolism, apoptosis, DNA repair, and cellular aging [43]. Moreover, numerous literature reports indicate the involvement of sirtuins in inflammatory processes [44], oxidative stress [45], mitochondrial functions [46], and carcinogenesis [47]. The diverse tissue-specific localization of individual sirtuins predisposes them to involvement in the pathogenesis of specific organ-related disorders [48,49]. Investigating the interplay between sirtuins, CR, and pancreatic β-cells is of particular importance, as it may provide new insights into the molecular mechanisms underlying glucose homeostasis and metabolic health. Sirtuins act as NAD^+^-dependent regulators of cellular metabolism, stress responses, and longevity pathways, many of which are directly relevant to β-cell function and survival. CR, in turn, is a well-established intervention that enhances sirtuin activity and improves metabolic efficiency [50]. Since pancreatic β-cell dysfunction is a central event in the pathogenesis of diabetes, understanding how CR-induced modulation of sirtuins influences β-cell physiology could uncover novel strategies for preserving β-cell function, delaying disease onset, and developing targeted therapeutic approaches.

Despite substantial progress in understanding the molecular biology of sirtuins, several important questions remain unresolved. It remains unclear which sirtuins are the principal mediators of the beneficial effects of caloric restriction in pancreatic β-cells, whether their functions are complementary or partially redundant, and to what extent the findings from experimental models can be translated to human physiology. Moreover, although individual sirtuins are discussed separately in the following sections, they should not be considered as isolated regulators of β-cell biology. Rather, they form an interconnected regulatory network in which nuclear sirtuins (primarily SIRT1, with additional contributions from SIRT6 and SIRT7) regulate gene expression, chromatin remodeling, and inflammatory signaling, whereas mitochondrial sirtuins (SIRT3, SIRT4, and SIRT5) orchestrate mitochondrial metabolism by controlling oxidative phosphorylation, antioxidant defense, amino acid and fatty acid metabolism, and ATP production required for glucose-stimulated insulin secretion. Therefore, the following sections critically summarize the current evidence for each member of the sirtuin family while highlighting common mechanisms, unresolved controversies, and remaining knowledge gaps relevant to pancreatic β-cell function and caloric restriction.

### 3.1. SIRT1

Research on the role of sirtuins in pancreatic β-cells is currently being conducted in pancreatic β cell lines, MIN6 (mouse), and INS1 (rat), in primary islets isolated from C57BL/6 mice, Sprague–Dawley (SD), and Wistar rats [14], in SIRT1 knockout mice [51] and in pancreatic β-cell-specific SIRT1-overexpressing (BESTO) transgenic mice [51,52]. Furthermore, Deng and colleagues employed an experimental model of rats with induced type 2 diabetes [53].

Sirtuin 1 is the most widely researched member of the sirtuin family. In relation to its function as a pivotal mediator of metabolism during periods of caloric restriction, SIRT1 can be utilized as a calorie restriction mimetic, thereby facilitating health maintenance without the necessity of behavioral calorie restriction. Maynihan et al. [52] observed that SIRT1 expression was confined to pancreatic islets, with no detectable presence in exocrine regions of the pancreas, indicating a potential involvement of SIRT1 in endocrine pancreatic activity. Wu et al. [54] observed the expression of SIRT1 in endocrine progenitors following the dynamic pattern of neurogenin3+ (NGN3) during pancreatic development, reaching its peak at E15.5 and declining by birth [54]. While others reported that SIRT1 is abundant in mature β-cell nuclei, Wu observed minimal expression in developing β-cells, suggesting distinct expression profiles in immature versus mature states. Furthermore, SIRT1 activation with SRT1720 has been demonstrated to enhance endocrine differentiation in pancreatic rudiments, as indicated by increased progenitor numbers, β-cell mass, and expression of differentiation markers [48]. Within the islets, the mature β-cells exhibited a faint, punctate nuclear SIRT1 signal, accompanied by a more diffuse cytoplasmic distribution. However, the intensity of the signal differed among individual cells [52]. Similar results were obtained by Chen et al. [14] in pancreatic islet β-cells isolated from Sprague–Dawley rats. What is interesting, however, is that the expression of SIRT1 was significantly increased in response to caloric restriction, which protects β-cells from apoptosis and regulates gluconeogenesis and glycolysis to maintain glucose homeostasis, whereas it decreased in response to high-fat and high-fructose diets. Chen et al. [14] and Deng et al. [53] employed a type 2 diabetic rat model to investigate the impact of caloric restriction on SIRT1 expression and the apoptosis of islet beta cells. CR has been demonstrated to enhance SIRT1 expression in islet β-cells and reduce the apoptosis ratio of these cells in rodent models. However, further elucidation is required to ascertain the causal relationship between SIRT1 expression and the apoptosis of islet β-cells.

Increased β-cell apoptosis plays a pivotal role in the loss of β-cell mass and the subsequent onset of type 2 diabetes [55]. The regenerative processes that determine their survival and functional capacity are also important. Two primary strategies have been identified for regenerating endogenous β-cells: stimulating the replication of existing β-cells and generating new β-cells from other cell types [56]. While the proliferation of β-cells declines after birth, the administration of growth factors has been demonstrated to enhance this process in rodents. Neogenesis, or transdifferentiation from non-β cells, requires regulatory factors; however, the molecular mechanisms underlying this process remain poorly understood. SIRT1 involvement in the differentiation, regeneration, and proliferation of stem cells has also previously been reported [57]. Wu et al. [54] demonstrated that SIRT1 activation with SRT1720 promotes β-cell regeneration by enhancing the activation and differentiation of endocrine progenitors derived from pancreatic ductal cells in two different models: neonatal rats treated with streptozotocin (STZ) and a pancreatic rudiment culture model. This process may be achieved through AMPK signaling-mediated regulation of fatty acid oxidation [54]. These complementary mechanisms are essential for understanding disease progression and potential therapeutic strategies.

SIRT1 has been reported to influence endocrine signaling involved in glucose and lipid homeostasis through the regulation of transcription factors, including forkhead transcription factor 3a (*FOXO3a*), glucose transporter 4 (GLUT4), peroxisome proliferator-activated receptor gamma (PPARγ), and its coactivator PGC-1α [14]. Moreover, SIRT1 has been demonstrated to act as a positive regulator of insulin secretion in pancreatic β-cells. It was hypothesized that this effect could result from repression of the uncoupling protein 2 (UCP2) gene, achieved by the direct binding of SIRT1 to the UCP2 promoter [51]. In β-cell lines with diminished SIRT1 expression via RNA interference (siRNA), UCP2 levels were augmented, concomitant with impaired insulin secretion. Increased UCP2 expression was found to be associated with a failure of the cells to elevate ATP levels following glucose stimulation. Conversely, downregulation of UCP2 restored insulin secretion in cells with reduced SIRT1 expression, confirming that UCP2 is a mediator of the defect in glucose-stimulated insulin release. Moreover, the deprivation of nutrients has been observed to induce UCP2 expression in the mouse pancreas, presumably as a consequence of a decline in pancreatic NAD (a niacin-derived metabolite) and a concomitant reduction in SIRT1 expression. In the absence of the SIRT1 gene, mice exhibited elevated levels of UCP2 [51]. Another study, conducted on mouse pancreatic β-cells (β-TC-6), has shown that exposure to perfluorooctane sulfonate (PFOS) inhibited silent information regulator 1 (SIRT1) activity and upregulated uncoupling protein 2 (UCP2) expression [58]. This upregulation was blunted in the presence of Ex-527, a SIRT1-specific inhibitor. The mitochondrial membrane potential (ΔΨm), as well as glucose-induced ATP production and Ca^2+^ influx, decreased under PFOS treatment. As a consequence, 48 h of PFOS exposure impaired glucose-stimulated insulin secretion [58].

On the other hand, Pinho et al. [59], using the β-cell-specific SIRT1 knockout mouse model (Pdx1Cre; Sirt1ex4F/F), found that the functionality of the β-cells was impaired, as evidenced by a reduction in glucose-stimulated insulin secretion. The defect was not attributable to diminished insulin expression; rather, it was concomitant with diminished expression of the glucose transporter Slc2a2/Glut2 and of the glucagon-like peptide-1 receptor (Glp1r), in addition to the marked downregulation of endoplasmic reticulum (ER) chaperones that participate in the unfolded protein response (UPR) pathway. In a counterintuitive finding, SIRT1-deficient mice did not develop hyperglycemia, and no changes in UCP2 expression were observed [59]. Still, the differences in experimental design could have contributed to the observed discrepancies.

Ramachandran et al. [60] highlighted the crosstalk between SIRT1 and microRNA in pancreatic β-cells. They reported that miR-9 and SIRT1 protein levels were actively regulated in vivo in β-islets during glucose-dependent insulin secretion. These data also demonstrated that miR-9 targets and regulates SIRT1 expression in insulin-secreting cells. This targeting was relevant in pancreatic β-islets, where a reduction in SIRT1 protein levels occurs when miR-9 expression is high, contributing to glucose-dependent insulin secretion [60]. What is interesting, is that the zinc-activated SIRT1-Pgc-1α axis takes part in glucose-stimulated insulin secretion in islets. Wang et al. [61] showed that zinc transporter Slc39a5 deficiency leads to significantly lower intracellular zinc levels. As a result, zinc deficiency downregulated *Sirt1* activity and *Pgc-1α* expression, which further affected Glut2 expression and mitochondrial function. Consequently, Slc39a5 deficiency in β-cells attenuates insulin secretion [61].

Wu et al. [54] assessed the roles of SIRT1 in islet β-cells under conditions of lipotoxicity. Using a rat model infused with 20% Intralipid for 24 h, they demonstrated that both SIRT1 mRNA and protein expression were markedly reduced in isolated islets. Similarly, long-term exposure to palmitate decreased SIRT1 expression in cultured INS-1 cells and isolated rat islets; this effect was prevented by treatment with resveratrol, a known SIRT1 agonist. Resveratrol also restored glucose-stimulated insulin secretion impaired by palmitate, whereas this protective effect was abolished by EX527, a specific SIRT1 inhibitor. Moreover, inhibition of SIRT1 activity using EX527 or SIRT1 knockdown suppressed insulin promoter activity and concomitantly reduced mRNA expression of insulin, v-maf musculoaponeurotic fibrosarcoma oncogene homolog A (*MafA*), and NK6 homeodomain 1 (*NKX6.1*). In contrast, activation of SIRT1 by resveratrol or SIRT1 overexpression counteracted palmitate-induced inhibition of insulin transcriptional activity. SIRT1 overexpression further enhanced pancreatic and duodenal homeobox 1 (PDX1)-driven insulin promoter activity and completely abolished the repressive effect of forkhead box O1 (*FOXO1*) on insulin transcription. Additionally, resveratrol reversed palmitate-induced *FOXO1* nuclear translocation. In a recent study, Desai et al. [62] provided compelling evidence that activation of SIRT1 attenuates the fat-induced decrease in β-cell function in vivo. The authors used the intravenous infusion of Wistar rats with either saline or oleate, with or without the SIRT1 activator resveratrol. Moreover, the same experimental protocol was employed in β-cell-specific SIRT1 overexpressing (BESTO) mice and wild-type littermates. Lipid infusion resulted in a significant decrease in β-cell function, as expected, in both animal models. SIRT1 activation, which did not alter β-cell function in the absence of fat, resulted in partial protection from the fat-induced decrease in β-cell function [62]. The conclusions were derived from the results of glucose-stimulated insulin secretion, an insulin sensitivity index, and a disposition index. Moreover, other authors established an in vitro model of β-cell dysfunction by exposing INS-1 cells to palmitate (PA), including control, PA-treated, and PA combined with nicotinamide mononucleotide (NMN) or pathway modulators [63]. Compared with the control group, PA treatment markedly impaired insulin secretion and reduced the expression of proteins within the NAD^+^/AMPK/SIRT1/HIF-1α pathway. Supplementation with NMN restored pathway protein expression through NAD^+^ activation and significantly improved insulin secretory function. Findings obtained with HIF-1α and AMPK activators or inhibitors further substantiated these results [64]. Collectively, the researchers demonstrated that NMN counteracted PA-induced downregulation of the NAD^+^/AMPK/SIRT1/HIF-1α pathway, thereby alleviating β-cell dysfunction [63]. Pancreatic β-cell lipotoxicity and glucolipotoxicity may contribute to pancreatic β-cell senescence [64]. Previously, Zhu et al. [64] demonstrated that 7-ketocholesterol (7-KC), the main oxidation product of cholesterol, significantly increased SA-β-gal activity, promoted G0/G1 arrest, DNA damage, and interleukin-1β expression in MIN6 cells, and significantly inhibited insulin synthesis. This study indicated that 7-KC led to pancreatic β-cell senescence, possibly by regulating activation of the SIRT1/CDK4–Rb—E2F1 signaling pathway [64].

**Figure 2:**
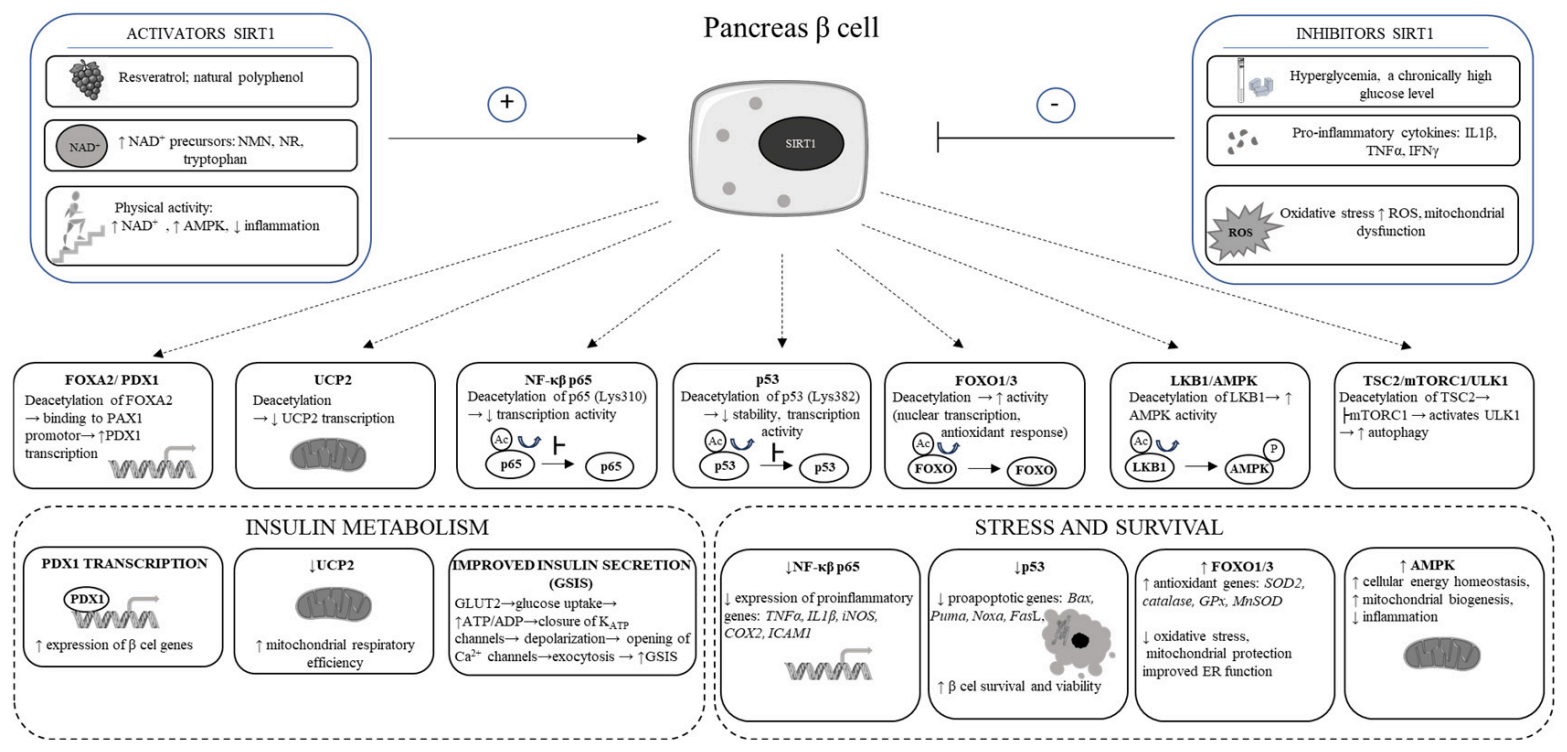
Schematic role of SIRT1 in pancreatic β-cells. → activation/stimulation, ├ inhibition, ↑ increase, ↓ decrease. Created by the authors using Microsoft PowerPoint with graphical elements from Servier Medical Art (https://smart.servier.com), licensed under the Creative Commons Attribution 4.0 International License (CC BY 4.0).

β-Cell sirtuin 1 expression is subject to regulation by a variety of endogenous and exogenous substances, reflecting its central role in cellular pathways, including the incretin hormone glucagon-like peptide 1 (GLP-1) [65], gamma-aminobutyric acid (GABA) [66], and fibroblast growth factor 1 (FGF1) [23]. Figure 2 summarizes the role of SIRT1 in β-cells. A comprehensive review of the extant literature reveals compelling evidence that substantiates the protective function of SIRT1 in the context of β-cell physiology. However, the majority of extant studies have been conducted in rodent or in vitro models, and the relative contribution of SIRT1 to β-cell recovery during caloric restriction in humans remains to be established.

### 3.2. SIRT2

SIRT2 gene expression has been confirmed in pancreatic β-cells in animal and human models, and in pancreatic cell lines [67,68]. Zhou et al. [67] observed the abundance of SIRT2 in normal islets obtained from male Sprague–Dawley (SD) rats. Interestingly, its mRNA level was more than twice that of SIRT1. As in other tissues, SIRT2 was mainly located in the cytoplasm of islet cells. Worthman et al. [68], using immunofluorescence staining, identified SIRT2 in β-cells in the mouse pancreas and human islets, and the level was age-dependent. SIRT2 protein was elevated in islets from 1-year-old mice compared with 1-month-old mice [68]. In MIN6 cells, SIRT2 levels have been shown to be regulated depending on metabolic conditions—its expression decreases in response to chronic hyperglycemia and oxidative stress [69]. Studies have shown that SIRT2 plays an important regulatory role in pancreatic β-cells, impacting their proliferation, metabolic adaptation, and functional homeostasis [68,69]. In a recent study, Wortham et al. [68] demonstrated that selective inactivation of SIRT2 in mouse β-cells resulted in increased proliferation under hyperglycemic conditions while maintaining feedback control over β-cell mass. These findings suggest that SIRT2 functions as a regulatory factor, limiting adaptive β-cell proliferation in response to excess glucose. Proteomic analysis of pancreatic islets lacking SIRT2 function or subjected to pharmacological blockade showed increased acetylation of enzymes involved in oxidative phosphorylation [68]. This finding indicates that SIRT2 normally deacetylates these enzymes and thereby suppresses increased oxygen consumption (O_2_) under hyperglycemic conditions [68]. At the transcriptomic level, Sirt2 inactivation has context-dependent effects on β-cells, with Sirt2 controlling how β-cells interpret hyperglycemia as a stress. There is evidence for the efficacy of the systemic administration of a glucagon-like peptide 1-coupled (GLP1-coupled), SIRT2-targeting antisense oligonucleotide in achieving β-cell SIRT2 inactivation and stimulating β-cell proliferation during periods of hyperglycemia [68].

In the context of secretory function, although direct data on SIRT2 and GSIS in β-cells remain limited, indirect evidence suggests that mitochondrial metabolic dysfunction and increased oxidation may negatively affect β-cell secretory response. For example, overexpression of the epithelial sodium channel alpha subunit (α-ENaC), a voltage-independent sodium ion channel, was associated with increased SIRT2 degradation in MIN6 β-cells, which correlated with impaired GSIS and endoplasmic reticulum stress [69]. Another study performed by Qian et al. [70] showed that SIRT2 interacts with the inflammatory microenvironment. Research has demonstrated that the downregulation of SIRT2 by miR-212-5p or exosomes secreted by M1 macrophages (M1-Exos) resulted in inhibition of the Akt/GSK-3β/β-catenin pathway. Decline in Akt phosphorylation, followed by an elevation in GSK-3β activity, caused a reduction in β-catenin levels, which play a pivotal role in modulating the actin cytoskeleton and the localization of insulin vesicles. These results indicate that SIRT2 is an important mediator in glucose-stimulated insulin secretion [70].

There is no single article (at least in the databases searched) that directly examines the effect of caloric restriction on SIRT2 in pancreatic β-cells. Taking into account that it has been demonstrated that SIRT2 responds to CR and oxidative stress [71], it can be deduced that under conditions of caloric restriction, increased levels of NAD^+^ and SIRT2 activation in pancreatic β-cells would promote FOXO3 deacetylation and the activation of protective pathways (e.g., antioxidant). This, in turn, could contribute to increased β-cell resistance to metabolic stress and delay their aging. Consequently, SIRT2-FOXO3 modulation in the context of CR represents a promising research avenue in the field of pancreatic β-cell function and survival.

### 3.3. SIRT3

Expression of SIRT3 has previously been detected in cats [72], mice [73], human [74] pancreatic β-cells, and the rat insulinoma cell line (INS-1) [74]. While SIRT3 was primarily thought of as a mitochondrial-localized member of the sirtuin family, a small fraction was also found in the nucleus of INS 832/13 cells [75]. It is suggested that SIRT3 expression appears to be variable both between species and between individuals [72]. As shown in the work of Zhang Y et al. [76], during fasting or after exposure to low glucose concentration, the SIRT3 protein level increased in the rat pancreatic islets [76]. As expected, the opposite effect was observed after high glucose treatment; both SIRT3 mRNA and protein expression levels displayed significant decreases [76].

SIRT3 has been shown to exert an anti-inflammatory effect [77]. As chronic inflammation and mitochondrial dysfunction are key factors mediating pancreatic β-cell impairment in T2DM, Caton and co-workers investigated the potential role of SIRT3 activation in protecting β-cell function in diabetic conditions [74]. The expression of both SIRT3 mRNA and protein was significantly reduced in the rat insulinoma cell line (INS-1) in response to proinflammatory stimuli, such as TNFα and IL-1β, which are elevated in diabetic individuals [74]. Consequently, a significant decrease in SIRT3 mRNA expression was observed in islets from diabetic subjects compared with their respective control groups [74]. Furthermore, studies have demonstrated that SIRT3 knockdown in INS-1 cells impairs insulin secretion and mRNA levels of several factors involved in insulin synthesis, such as MafA and duodenal homeobox 1 (PDX1), underscoring the critical function of SIRT3 in maintaining β-cell function. The results of the study demonstrated that SIRT3 plays a critical role in maintaining the mass of β-cells. Suppression of SIRT3 led to a substantial increase in INS1 apoptosis, suggesting a pivotal function for SIRT3 in preserving β-cell mass [78].

As previously stated, lipid accumulation is a primary risk factor for β-cell dysfunction. In a study conducted by Kim et al. [79], it was reported that SIRT3 protects against palmitate-induced β-cell dysfunction in NIT1 cells [79]. Specifically, SIRT3 overexpression significantly reduced palmitate-induced apoptosis, as evidenced by the decreased levels of caspase-3 activity compared to control cells. Notably, palmitate has been observed to impede ATP production and insulin secretion, while SIRT3 enhancement has been shown to increase both ATP and glucose-stimulated insulin release. Consistent outcomes were also observed in rat islets overexpressing SIRT3, further substantiating the pivotal function of SIRT3 in β-cell function. Furthermore, given the established role of palmitate-induced endoplasmic reticulum stress in β-cells in promoting apoptosis and diabetes, a notable decrease in the mRNA expression of *ATF4*, *GRP94*, and *FKBP11* (factors commonly activated by ER stress) was observed in SIRT3-overexpressing cells [79].

Similar observations were made by Zhang [80], who reported that SIRT3 expression was significantly reduced in NIT1 and INS1 cells exposed to palmitate. The administration of palmitate led to a substantial decline in the viability and insulin secretion of β-cells while concurrently promoting the formation of malondialdehyde (MDA). However, SIRT3 expression in NIT1 and INS1 cells led to the opposite outcomes, resulting in increased insulin secretion, decreased β-cell apoptosis, and reduced expression of ER stress-associated genes. Furthermore, SIRT3 expression has been shown to impede calcium influx and the hyperacetylation of glucose-regulated protein of 78 kD (GRP78). SIRT3 knockdown effectively enhanced the upregulation of phospho kinase-like endoplasmic reticulum kinase (pERK), inositol-requiring enzyme-1 (IRE1), activating transcription factor 6 (ATF6), and C/EBP homologous protein (CHOP) induced by palmitate, and promoted palmitate-induced β-cell apoptosis and dysfunction [80].

It has been suggested that in addition to lipotoxicity, glucotoxicity, oxidative stress, endoplasmic reticulum stress, and inflammation, β-cell dedifferentiation has emerged as a new player in β-cell failure, including apoptosis and senescence [81]. Dedifferentiated β-cells become progenitor-like cells or transdifferentiate into non-β pancreatic cells, contributing to the progression of T2D. Previous studies have demonstrated that during this process, vital gene markers for mature β-cell function were downregulated, while progenitor cell markers and “disallowed” genes were upregulated [78,81]. Cao provided compelling evidence that SIRT3 prevented β-cell dedifferentiation by inhibiting autotaxin (ATX) expression and the upregulation of lysophosphatidic acid (LPA), both of which contributed to pancreatic β-cell dedifferentiation. Results obtained by Nie Y et al. [78] are partly consistent with those of Cao H et al. [81] A study showed that SIRT3 induces β-cell dedifferentiation by affecting the amount and deacetylation of FOXO1 and the subsequent activity, which might be another mechanism of β-cell failure. These findings highlight the protective role of SIRT3 in maintaining β-cell function under lipotoxic conditions, such as those associated with diabetes. Furthermore, SIRT3 is associated with mitofusin2 (Mfn2) signaling, which has been implicated in the maturation and functionality of β-cells. The suppression of SIRT3 in Mfn2-overexpressing cells has been demonstrated to result in a reduction in insulin secretion [82].

Taking into account recent reports indicating the influence of protein acetylation in energy metabolism [76,83,84], Zhang et al. [76] performed the first large-scale profiling of acetylome in rat islets, showing that almost all enzymes in core metabolic pathways related to insulin secretion were acetylated, especially those involved in fatty acid β-oxidation (FAO), including the key subunit-trifunctional enzyme subunit alpha (ECHA). Acetylation decreased the stability and ability of ECHA to promote FAO. Overexpression of SIRT3 prevented ECHA degradation by decreasing its acetylation level in β-cells. SIRT3 expression increased in rat islets when exposed to low glucose or during fasting. However, SIRT3 overexpression in islets markedly decreased palmitate-potentiated insulin secretion. In contrast, islets from SIRT3 knockout mice secreted more insulin and exhibited the opposite effect on FAO. ECHA overexpression partially reversed the insulin hypersecretion elicited by SIRT3 deficiency [76].

Interestingly, recent evidence suggests that prenatal testosterone exposure caused suppression of SIRT3 expression and activation of oxidative stress in pancreatic beta cells of aged female offspring [73]. Moreover, overexpression of SIRT3 in islets isolated from female offspring treated with prenatal testosterone normalized the oxidative stress level, restored cyclic AMP, and ATP generation, which finally improved glucose-stimulated insulin secretion in beta cells [73].

Naatz A et al. [75] provided evidence that SIRT3 plays a primary role in regulating β-cell expression of *Gadd45α* and DNA repair in β-cells exposed to nitric oxide. For the first time, authors observed SIRT3 not only in the mitochondria-containing cytosolic fraction, but also, interestingly, in the nuclear fraction in INS 832/13 cells [75]. It has been shown that siRNA knockdown of SIRT3 attenuates nitric oxide-stimulated Gadd45α mRNA accumulation in both wild-type and SIRT3−/−INS 832/13 cells as well as isolated rat islets, and that SIRT3 inhibition increases FOXO1 acetylation and attenuates DNA repair in response to nitric oxide [75].

Although SIRT3 is widely recognized as a key regulator of mitochondrial homeostasis, it remains unclear whether its activation alone is sufficient to improve β-cell function or whether it primarily acts in concert with other mitochondrial sirtuins during CR.

### 3.4. SIRT4

The mitochondrial localization of endogenous SIRT4 in the mouse insulinoma β-cell line MIN6 and INS1 has been verified [5,85]. Anderson et al. [86] demonstrated that pancreatic islets were among the highest SIRT4-expressing tissues. SIRT4, a mitochondrial ADP-ribosyltransferase, has been shown to modify glutamate dehydrogenase (GDH), thereby affecting insulin secretion and amino acid metabolism in the pancreas [85]. It has been demonstrated that GDH facilitates the metabolism of glutamate and glutamine, thereby generating ATP, which in turn promotes insulin secretion. The loss of SIRT4 in insulinoma cells has been shown to activate GDH, thereby upregulating amino acid-stimulated insulin secretion. A comparable effect was observed in pancreatic β-cells from mice deficient in SIRT4 or following a dietary regimen of CR. Furthermore, GDH from SIRT4-deficient or CR mice was insensitive to phosphodiesterase, an enzyme that cleaves ADP-ribose, suggesting the absence of ADP-ribosylation. These results indicate that SIRT4 performs a regulatory function in β-cell mitochondria, modulating GDH activity via ADP-ribosylation, thereby affecting insulin secretion in response to amino acids. The observed effects of this regulatory process are mitigated during caloric restriction [85]. Recent studies have demonstrated that SIRT4 catalyzes the removal of several newly identified lysine acyl modifications, including methylglutaryl (MG)-lysine, hydroxymethylglutaryl (HMG)-lysine, and 3-methylglutaconyl (MGc)-lysine [87]. Through the sequential removal of these modifications, SIRT4 participates in regulating leucine oxidation, a key allosteric activator of glutamate dehydrogenase (GDH) [87]. Moreover, leucine has long been known as a potent insulin secretagogue [80]. The study performed by Anderson et al. [86] demonstrated that dysregulated leucine metabolism in SIRT4 knockout mice leads to elevated basal and nutrient-stimulated insulin secretion, ultimately progressing to glucose intolerance and insulin resistance. These observations establish a definitive enzymatic function for SIRT4, reveal a regulatory mechanism governing branched-chain amino acid flux, and highlight SIRT4 as a key metabolic regulator that preserves appropriate insulin secretion and glucose homeostasis during aging.

Ahuja et al. [5] identified two additional targets for SIRT4: insulin-degrading enzyme (IDE) and ADP/ATP translocases (ANT2/3). Deletion of SIRT4 decreased ATP levels, and overexpression of SIRT4 increased ATP levels [87]. In addition, SIRT4 can interact with insulin-degrading enzyme (IDE) and ADP/ATP translocase 2/3 (ANT2/3) to synergistically inhibit insulin secretion [88]. Depletion of SIRT4 from insulin-producing INS-1E cells resulted in increased insulin secretion in response to glucose [5]. Knowledge of the role of SIRT4 in β-cell metabolism provides new insights into the potential development of therapeutic agents targeting its enzymatic activities.

### 3.5. SIRT5

In a study performed by Ma et al. [89], it was demonstrated that SIRT5 is expressed and functionally active in the MIN6 and INS-1 cell lines, and it influenced insulin secretion and β-cell proliferation. The silencing of SIRT5 using siRNAs resulted in elevated levels of insulin secretion in response to glucose stimulation (1 mM vs. 20 mM) and augmented β-cell proliferation in both lines. Furthermore, in the same models, SIRT5 was found to play a role in regulating the expression of the transcription factor PDX1. It is known that PDX1 is the earliest tissue-selective transcription factor, essential to the formation of all pancreatic cell types and the activity of adult islet β-cells. Furthermore, the results of this study demonstrated that the expression of SIRT5 is inversely correlated with PDX1, suggesting a potential regulatory relationship between these two factors.

According to the findings of Wang et al. [90], SIRT5 overexpression in the NIT-1 β-cell line under glucolipotoxic conditions (high glucose combined with palmitate) markedly reduces apoptosis and improves the glucose-stimulated insulin response. This protective effect is accompanied by decreased caspase-3 activity and reduced lipid peroxidation, as well as preservation of the anti-apoptotic proteins BCL-2 and BCL-XL. These observations indicate that SIRT5 may exert a cytoprotective role in β-cells exposed to metabolic stress.

It is important to note that although the relationship with caloric restriction has not been directly studied in these lines, the data provide a strong foundation for the hypothesis that SIRT5 modulation in β-cells may be indirectly related to metabolic mechanisms activated by CR (e.g., through changes in the deacetylation of mitochondrial enzymes and transcriptional regulators).

### 3.6. SIRT6

In 2006, Mostosławski et al. [91] observed that SIRT6 loss leads to abnormalities in mice that overlap with aging-associated degenerative processes. Since then, a series of studies have been conducted confirming the involvement of SIRT6 in genome stability and metabolic homeostasis, linking DNA repair, aging, and energy regulation [92,93,94].

The role of SIRT6 in glucose metabolism is underscored by its pivotal function in enhancing glucose-stimulated insulin secretion and ATP production in pancreatic β-cells [95]. Mechanistically, these effects may be linked to mitochondrial dysfunction observed in SIRT6-deficient β-cells, including impaired mitochondrial function, altered Ca^2+^ dynamics, and reduced oxygen consumption [95]. Deletion of SIRT6 has been shown to increase β-cell apoptosis and compromise GSIS in MIN6 cells, whereas SIRT6 overexpression conferred protection against palmitate-induced β-cell dysfunction and apoptosis [95,96]. Moreover, Qin et al. [97] demonstrated that SIRT6 is essential for β-cell function and survival in a mouse model. While SIRT6 deficiency did not alter the overall endocrine morphology, β-cell mass, or insulin content, it resulted in glucose intolerance and impaired GSIS. β-Cell-specific deletion of SIRT6 recapitulated these defects, indicating a cell-autonomous effect. Mechanistically, loss of SIRT6 led to an increased acetylation of histone H3 at lysines 9 and 56 (H3K9Ac, H3K56Ac), and enhanced RNA polymerase II recruitment to the promoter of thioredoxin-interacting protein (TXNIP) [97]. Given that TXNIP expression inversely correlates with GSIS and that its overexpression suppresses insulin secretion [98], these findings suggest that SIRT6 maintains β-cell function by epigenetically repressing TXNIP. It is also very important to note that SIRT6 has emerged as a critical guardian of genomic stability, linking cellular energy status to the maintenance of chromatin integrity and DNA repair mechanisms [99]. Because SIRT6 activity is dependent on NAD^+^ availability, the elevation of intracellular NAD^+^ levels induced by caloric restriction or NAD^+^ precursor supplementation may enhance its protective functions. Activated SIRT6 promotes telomeric chromatin maintenance, facilitates the repair of DNA damage, and limits replicative stress, thereby reducing genomic instability and delaying the onset of cellular senescence. Through these mechanisms, SIRT6 may contribute to the long-term preservation of pancreatic β-cell viability and functional capacity under metabolic stress conditions. In the context of nuclear–mitochondrial crosstalk, NAD^+^-dependent activation of SIRT6 represents an important mechanistic link between metabolic adaptation, epigenetic regulation, and cellular longevity, supporting the maintenance of β-cell homeostasis during aging and caloric restriction [99].

Additionally, β-cell-specific SIRT6-deficient mice exhibited increased susceptibility to streptozotocin-induced β-cell apoptosis [97]. Recently, Park et al. [100] investigated the role of SIRT6 during β-cell aging. It was observed that SIRT6 protein levels were decreased in pancreatic β-cells during aging. In addition, pancreatic β-cell-specific *Sirt6* transgenic (TgSIRT6) mice were characterized by less DNA damage and cell death, including apoptosis, necroptosis, and pyroptosis, in β-cells than the control mice. Moreover, transgenic mice exhibited an increase in both total islet area and pancreatic β-cell mass compared with the control animals. Correspondingly, TgSIRT6 mice demonstrated improved glucose tolerance and enhanced glucose-stimulated insulin secretion. Mechanistically, SIRT6 negatively regulated RRAD and GEM-like GTPase 2 (REM2), an endogenous inhibitor of high-voltage-activated calcium channels. Knockdown of *Rem2* in INS-1 cells partially mitigated DNA damage, lipid peroxidation, and cell death induced by SIRT6 deficiency or palmitic acid treatment. Consistently, β-cell-specific Rem2 knockout mice displayed reduced DNA damage and lower rates of β-cell apoptosis relative to the controls. These findings indicate that SIRT6 functions as a critical anti-aging factor in pancreatic β-cells [93]. SIRT6 also mediates the beneficial effects of caloric restriction (CR), a well-established intervention known to delay the onset of age-related diseases, including diabetes and cardiovascular disorders [93]. Deletion of SIRT6 abrogated CR-induced lifespan extension, indicating that SIRT6 is essential for CR-mediated longevity [93]. Mechanistically, CR-activated SIRT6 suppresses NF-κB signaling, thereby contributing to delayed aging. Furthermore, transgenic mice overexpressing SIRT6 exhibit multiple phenotypes reminiscent of CR, including reduced body weight, enhanced metabolic activity, and decreased serum levels of cholesterol, adipokines, insulin, and glucose [101]. Bian et al. [102] assessed the role of SIRT6 in glucolipid metabolism in Sprague–Dawley rats as well as in BRL 3A and INS-1 cell lines under overnutrition and starvation conditions. The mRNA and protein expression levels of SIRT6 in the liver and pancreas of the high-fat diet group were low, but these levels were high in the starvation group. Opposite results were observed in cell lines. In the INS1 cells, the mRNA expressions of *SIRT6*, *AMPK*, and *mTOR*, the related proteins SIRT6, p-AMPKα/AMPKα, and the mTORC1 complex-related factors (p-mTOR/mTOR, p-Raptor/Raptor, and Rheb) were significantly increased; the mRNA expressions of metabolic enzymes (Gck and Pck1) were significantly increased; and the activity of CCK-8 was significantly decreased in the high glucose group. Recombinant SIRT6 mitigated the reduction in AMPKα and the elevation of mTORC1 (comprising mTOR, Raptor, and Rheb) induced by overnutrition. Knockdown of SIRT6 via siRNA counteracted the glucolipid metabolic disturbances triggered by AMPK activation or mTOR inhibition, but did not affect those caused by liver X receptor activation. Overall, these findings indicate that SIRT6 modulates glycolipid metabolism in the liver and pancreas under conditions of overnutrition and starvation by regulating the AMPKα–mTORC1–SREBP1c pathway, independently of LXR [102].

### 3.7. SIRT7

SIRT7 is emerging as an important regulator of metabolic and epigenetic programs in metabolic tissues [103]. In addition, current evidence indicates that research on the role of SIRT7 in pancreatic β-cells is actively progressing. A dedicated project titled “Role of SIRT7 in the Pancreatic Beta Cells”, funded by the National Institute of Diabetes and Digestive and Kidney Diseases (NIDDK/NIH), is being conducted under an NIH R01 mechanism [104]. The study aims to define whether SIRT7 regulates glucose-stimulated insulin secretion and protects β-cells from metabolic and inflammatory stressors associated with overnutrition. As the project is ongoing, definitive mechanistic conclusions regarding SIRT7’s involvement in β-cell responses to caloric restriction remain forthcoming; however, its scope underscores the growing scientific interest in SIRT7 as a potential mediator of β-cell resilience and metabolic adaptation. Compared with other family members, SIRT7 has received relatively little attention and its contribution to β-cell adaptation during caloric restriction remains largely unexplored.

## 4. Sirtuin Activators as Therapeutic Strategies for Pancreatic β-Cell Dysfunction

Recent evidence indicates that pharmacological and nutritional activation of sirtuins is a promising therapeutic strategy for preserving pancreatic β-cell function under metabolic stress [62,105]. Unlike genetic or mechanistic modulation of sirtuin expression, the use of sirtuin activators aims to restore β-cell homeostasis by enhancing endogenous cytoprotective pathways compromised in diabetes, aging, and nutrient overload. Among the direct sirtuin activators, resveratrol remains the most extensively studied compound in the context of pancreatic β-cell dysfunction [106]. Resveratrol (3,5,4′-trihydroxystilbene) is a polyphenolic compound found, among other places, in grape skins that has been shown to increase lifespan in various organisms, promoting effects similar to those induced by caloric restriction [107]. Resveratrol-induced activation of SIRT1 has been proposed as a coordinated metabolic strategy acting both in the liver and pancreatic β-cells to promote systemic glucose homeostasis, highlighting a unified regulatory role of SIRT1 across key metabolic tissues [107]. Experimental evidence subsequently supported this concept. It has been demonstrated that INS-1E cells and human islets treated with resveratrol resulted in marked potentiation of glucose-stimulated insulin secretion and protection of β-cells against oxidative stress-induced apoptosis. These effects were associated with enhanced SIRT1 activity and reduced activation of pro-apoptotic signaling pathways. Moreover, the upregulation of key genes involved in β-cell function, including *Glut2*, glucokinase, *Pdx-1*, *Hnf-1α*, and *Tfam*, was observed [108]. Importantly, resveratrol-mediated improvements in β-cell function were observed independently of systemic insulin sensitivity, suggesting a direct protective action at the level of the pancreatic islet [108]. Additionally, resveratrol influences the membrane potential of β-cells by modulating the K(ATP) and K(V) channels, resulting in membrane depolarization and increased insulin release [109]. Furthermore, Rouse, M et al. [110] demonstrated that resveratrol functions as a phosphodiesterase inhibitor, increasing cAMP levels and amplifying signaling pathways that support β-cell function and survival. In vivo models have shown that chronic resveratrol supplementation protects β-cells from oxidative and inflammatory stress, thereby restoring the ultrastructure of pancreatic islets and enhancing glycemic control in rat models of diabetes [111]. Recent studies performed by González-Blanco et al. [112] indicate that resveratrol reduces amylin aggregation, thereby alleviating mitochondrial and ER stress in β-cells. This finding is particularly significant in the context of glucotoxicity and the progression of pancreatic islet dysfunction [112]. Another study suggests that the therapeutic effects of polyphenolic sirtuin activators may extend beyond classical NAD^+^–SIRT signaling and involve the modulation of novel biomarkers associated with β-cell dysfunction and diabesity. In this context, a study conducted by Rehman et al. [106] identified secreted frizzled-related protein 4 (SFRP4) as a key inflammatory biomarker linking obesity, insulin resistance, and impaired β-cell function. SFRP4 has previously been implicated in defective insulin exocytosis and is considered an early marker of β-cell stress in type 2 diabetes. In a high-fat diet mouse model, supplementation with resveratrol and quercetin significantly reduced SFRP4 expression, accompanied by the attenuation of systemic inflammation and improvement of metabolic parameters. Molecular docking analyses further suggested a direct interaction of these polyphenols with SFRP4-related signaling components, supporting a mechanistic basis for their regulatory effects. Although the study did not directly assess sirtuin activity in pancreatic islets, the findings highlight an additional, sirtuin-independent layer through which resveratrol may preserve β-cell function by suppressing inflammatory mediators that interfere with insulin secretion. These data reinforce the concept that resveratrol exerts pleiotropic protective effects on β-cells, combining sirtuin activation with the modulation of emerging biomarkers such as SFRP4, thereby strengthening its potential as a therapeutic agent in diabesity-associated β-cell dysfunction [106]. Table 1 summarizes the molecular targets and role of resveratrol in pancreatic β-cells.

Despite these encouraging outcomes, the translational application of resveratrol is limited by its low bioavailability, rapid metabolism, and lack of selectivity for distinct sirtuin isoforms. To overcome these limitations, synthetic small-molecule SIRT1 activators such as SRT1720 and SRT2104 have been developed. SRT1720 was shown to mimic calorie restriction effects and improve metabolic parameters, including insulin sensitivity and mitochondrial respiration, in animal models of obesity and diabetes [113]. Wu et al. [54] demonstrated that pharmacological SIRT1 activation with SRT1720 significantly promotes β-cell regeneration in STZ-treated neonatal rats primarily by enhancing the differentiation of NGN3-positive endocrine progenitors derived from pancreatic ductal cells, rather than by the expansion of residual β-cells. Dynamic SIRT1 expression was observed in endocrine progenitors during β-cell regeneration and pancreatic development, and SRT1720 consistently increased progenitor differentiation in cultured pancreatic rudiments. Mechanistically, SIRT1 activation upregulated NGN3 through AMPK-dependent fatty acid oxidation, an effect abolished by AMPK inhibition, indicating that SIRT1 promotes β-cell restoration via AMPK-mediated FAO [54]. Moreover, SRT1720 has been shown to exert direct cytoprotective actions in pancreatic β-cells under hypoxic stress, as evidenced by improved cell viability and the reduced production of pro-inflammatory cytokines, including tumor necrosis factor-α (TNF-α), interleukin-6 (IL6), and reactive oxygen species (ROS), in hypoxia-exposed INS-1 cells [114]. Beyond its metabolic effects, SRT1720-mediated activation of SIRT1 induces histone deacetylation and chromatin remodeling, thereby regulating the expression of genes involved in mitochondrial quality control, antioxidant defense, autophagy, and cellular stress adaptation [115]. Through the deacetylation of transcription factors including FOXO proteins and PGC-1α, SIRT1 promotes transcriptional programs that enhance mitochondrial biogenesis, improve resistance to oxidative stress, and preserve β-cell identity and insulin secretory capacity [116]. Consequently, activation of SIRT1 by SRT1720 affects not only cellular energy metabolism but also epigenetic regulation and chromatin plasticity, enabling sustained adaptive responses to caloric restriction.

Although direct experimental studies assessing the effects of another selective SIRT1 activator SRT2104, in pancreatic β-cell lines such as INS-1 or MIN6 have not been reported to date, substantial mechanistic evidence supports its potential relevance to β-cell stress responses. Pharmacological activation of SIRT1 by SRT2104 has been shown to attenuate systemic and cellular inflammation through the suppression of NF-κB signaling and reduction in pro-inflammatory cytokine production, pathways critically implicated in cytokine-mediated β-cell dysfunction [117]. In addition, SRT2104 enhances antioxidant defenses and preserves mitochondrial function in multiple non-β cellular models, partly via SIRT1-dependent regulation of oxidative stress-responsive pathways, including NRF2 signaling and p53 deacetylation [118]. Importantly, clinical studies in patients with type 2 diabetes have demonstrated that SRT2104 is generally well-tolerated and exerts modest systemic metabolic effects, including improvements in lipid profiles; however, these trials did not show consistent improvements in glycemic control or circulating insulin levels [119]. The lack of clear glucose-lowering efficacy in humans may reflect limited target engagement at the level of pancreatic β-cells, pharmacokinetic variability, or insufficient treatment duration. Nevertheless, given that oxidative stress, inflammatory signaling, and mitochondrial dysfunction are central drivers of β-cell failure in diabetes, these clinical and preclinical findings collectively provide a strong mechanistic rationale for further β-cell-focused investigation of SRT2104 as a synthetic SIRT1 activator.

An alternative and increasingly explored strategy involves the indirect activation of sirtuins through the enhancement of intracellular NAD^+^ availability. NAD^+^ boosters, including nicotinamide riboside (NR) and nicotinamide mononucleotide (NMN), have been shown to restore NAD^+^ levels in metabolically stressed tissues and thereby augment sirtuin activity. As mentioned earlier, in pancreatic β-cells, restoration of NAD^+^ biosynthesis has been linked to improved insulin secretion, mitochondrial function, and resistance to glucolipotoxicity [120]. Experimental evidence, performed by Cercillieux A [25], indicates that pancreatic β-cells express nicotinamide riboside kinase 1 (NRK1), enabling the efficient conversion of NR into NAD^+^ within islets. Genetic disruption of NR utilization results in impaired glucose tolerance and defective insulin secretion under conditions of metabolic stress, such as high-fat diet feeding and aging, underscoring the importance of NAD^+^ homeostasis for β-cell function. While acute NR supplementation does not significantly enhance glucose-stimulated insulin secretion in isolated islets from young, metabolically healthy animals, these findings suggest that the beneficial effects of NR may be context-dependent and become more evident under conditions of β-cell stress. Clinical studies evaluating NR supplementation in humans further support this notion [121]. In randomized, placebo-controlled trials conducted in individuals with obesity but without overt diabetes, NR administration increased systemic NAD^+^ levels but did not significantly improve insulin secretion, C-peptide responses, or indices of β-cell secretory capacity [121]. These neutral effects on β-cell function may reflect limited target engagement at the level of pancreatic islets, insufficient treatment duration, or the absence of pronounced β-cell dysfunction in the studied populations. In pancreatic β-cell models, NMN supplementation has been shown to restore NAD^+^ levels and activate SIRT1, improving insulin secretion and mitochondrial function under metabolic stress conditions. For example, in INS1 cells exposed to palmitate, NMN ameliorated β-cell dysfunction through the NAD^+^/AMPK/SIRT1/HIF-1α pathway, restoring glucose-stimulated insulin secretion and reducing stress-induced signaling perturbations [63]. Similarly, in vivo studies in mice demonstrated that NMN increased NAD^+^ content and SIRT1 expression in pancreatic islets after severe injury, leading to enhanced β-cell survival, mitochondrial protection, and improved glucose homeostasis, effects that were abolished by pharmacological SIRT1 inhibition [122]. Evidence from in vitro cellular systems, isolated pancreatic islets, and rodent models consistently indicates that sirtuin activation and restoration of intracellular NAD^+^ availability enhance β-cell function, preserve glucose homeostasis, and improve insulin sensitivity. However, these encouraging findings have not been fully replicated in clinical trials. Several biological factors may underlie this discrepancy. First, age-related alterations in human NAD^+^ metabolism, including reduced nicotinamide phosphoribosyltransferase (NAMPT) activity and increased NAD^+^ degradation by CD38, may limit the ability of NAD^+^ precursors to restore intracellular NAD^+^ pools sufficiently and activate sirtuins in metabolically relevant tissues [123]. Second, pancreatic β-cells constitute only a small fraction of pancreatic tissue, and the tissue specificity of currently available sirtuin activators and NAD^+^ boosters is limited, raising concerns about whether therapeutically effective concentrations are achieved within pancreatic islets [124,125]. Furthermore, commonly used rodent models often depict early-stage or experimentally induced diabetes in young animals. However, patients with type 2 diabetes frequently exhibit advanced disease, which is characterized by chronic inflammation, mitochondrial dysfunction, prolonged glucolipotoxicity, β-cell dedifferentiation, and irreversible loss of β-cell mass. Under these conditions, activation of a single longevity-associated pathway may be insufficient to restore endocrine pancreatic function. Furthermore, most clinical studies have administered these interventions after metabolic dysfunction has already occurred. However, experimental evidence suggests that activating sirtuins may be more effective as a preventive strategy to preserve β-cell integrity than to reverse advanced dysfunction [125]. Substantial inter-individual variability in NAD^+^ metabolism, sirtuin expression, inflammatory status, and metabolic phenotype may contribute to heterogeneous therapeutic responses, highlighting the need for biomarker-guided patient stratification [126]. Taken together, these findings suggest that successful clinical translation will likely require more selective, tissue-targeted sirtuin modulators; improved strategies to enhance intracellular NAD^+^ availability; combination therapies that address multiple pathogenic mechanisms; and earlier therapeutic intervention in individuals at risk of β-cell failure.

It is also important to emphasize that several important gaps remain regarding the precise contribution of individual sirtuins to pancreatic adaptation during caloric restriction. Current evidence is predominantly focused on SIRT1, whereas the coordinated roles of other family members, including mitochondrial SIRT3 and SIRT4 as well as less extensively characterized isoforms such as SIRT2, SIRT5, and SIRT7, remain insufficiently understood. Absence of this evidence is identified as a current gap in the literature. Any discussion of a potential relationship is presented as a hypothesis rather than an established finding. In particular, the integration of nuclear and mitochondrial sirtuin pathways and their contribution to β-cell metabolic resilience, mitochondrial quality control, and long-term functional preservation requires further investigation. Moreover, it remains unclear whether sirtuin activation represents a causal mechanism underlying CR-induced β-cell protection or rather a downstream consequence of broader metabolic adaptations involving AMPK, mTOR, insulin/IGF-1 signaling, and NAD^+^ metabolism.

## 5. Conclusions and Future Perspectives

Sirtuins act as key molecular mediators of caloric restriction in the pancreas, orchestrating adaptive responses that preserve β-cell function, enhance mitochondrial performance, modulate metabolic signaling, and attenuate oxidative and inflammatory stress. Activation of SIRT1 and related isoforms via polyphenols (e.g., resveratrol), synthetic compounds (SRT1720, SRT2104), or NAD^+^ precursors (NMN, NR) recapitulates many of the beneficial effects of CR in preclinical models, including improved glucose-stimulated insulin secretion, β-cell survival, and metabolic homeostasis.

Furthermore, sirtuin activation has occasionally yielded conflicting or context-dependent outcomes, reflecting differences in isoform specificity, dosage, model systems, sex, age, or metabolic status. Translational application remains limited by bioavailability constraints, isoform nonspecificity, incomplete clinical validation, and potential off-target consequences of systemic NAD^+^ augmentation. Future research should therefore focus on tissue- and isoform-specific sirtuin modulators, advanced approaches integrating single-cell multi-omics and functional models, and well-designed translational and clinical studies. Such investigations will be essential to define the precise role of individual sirtuins and their interactions within nuclear–mitochondrial regulatory networks, ultimately enabling the development of targeted interventions aimed at preserving β-cell function, maintaining metabolic health, and promoting healthy longevity.

## Figures and Tables

**Table 1:** Molecular targets and mechanisms underlying the effects of resveratrol in pancreatic β-cells.

Molecular Target/Pathway	Mechanism	Biological Outcome	Reference
SIRT1	Reduced activation of pro-apoptotic signaling pathways, Increased ATP generation	Protection of β-cells against oxidative stress-induced apoptosis, Potentiation of GSIS	[107]
KATP channels, Kv channels	Blockade of KATP and Kv channels, increased Ca^2+^ influx	Enhanced glucose-stimulated insulin secretion (GSIS)	[109]
Phosphodiesterases (PDEs), cAMP pathway	Increased intracellular cAMP	Enhanced insulin secretion	[110]
hIAPP aggregation, proteostasis pathways	Inhibition of hIAPP aggregate formation and reduction in aggregate-induced toxicity	Preservation of β-cell survival and prevention of islet amyloidosis	[112]
SFRP4/Wnt signaling	Downregulation of SFRP4 and inflammatory mediators	Improved metabolic function	[106]

## Data Availability

No new data were created or analyzed in this study.

## Abbreviations

The following abbreviations are used in this manuscript:

CR	Caloric restriction
GSIS	Glucose-induced insulin secretion
FFAs	Free fatty acids
AMPK	AMP-activated protein kinase
T2DM	Type 2 diabetes mellitus
NAMPT	Nicotinamide phosphoribosyltransferase
NRK1	Nicotinamide riboside kinase 1
Ins1, Ins2	Insulin gene 1, 2
Iapp	Amylin
G6pc2	Glucose-6 phosphatase enzyme G6pc2
SIRT1–7	Sirtuins 1–7
FOXO3a	Forkhead transcription factor 3a (FOXO3a)
GLUT4	Glucose transporter 4
PPARγ	Peroxisome proliferator-activated receptor gamma
UCP2	Uncoupling protein 2
Glp1r	Glucagon-like peptide-1 receptor
α-ENaC	Epithelial sodium channel alpha subunit
PDX1	Duodenal homeobox 1
GDH	Glutamate dehydrogenase
SFRP4	Secreted frizzled-related protein 4
ROS	Reactive oxygen species
NR	Nicotinamide riboside
NMN	Nicotinamide mononucleotide
